# Indonesian Ginger (Bangle) Extract Promotes Neurogenesis of Human Neural Stem Cells through WNT Pathway Activation

**DOI:** 10.3390/ijms21134772

**Published:** 2020-07-05

**Authors:** Kazumi Hirano, Miwa Kubo, Yoshiyasu Fukuyama, Masakazu Namihira

**Affiliations:** 1 Molecular Neurophysiology Research Group, Biomedical Research Institute, The National Institute of Advanced Industrial Science and Technology (AIST), Ibaraki 305-8566, Japan; 2 Department of Pharmaceutics, Faculty of Pharmaceutical Sciences, Tokushima Bunri University, Tokushima 770-8514, Japan; miwa-k@ph.bunri-u.ac.jp (M.K.); fukuyama@ph.bunri-u.ac.jp (Y.F.)

**Keywords:** neural stem cells, human neurogenesis, *Zingiber purpureum*, Banglene, WNT signaling, histone deacetylase

## Abstract

Indonesian ginger (*Zingiber purpureum* Rosc.), also known as Bangle, exhibits neurotrophic effects on cultured murine cortical neurons and in the adult mouse brain, but the underlying mechanisms remain unknown. Here, using human fetal neural stem cells (hfNSCs) as a model system for in vitro human neurogenesis, we show that Bangle extracts activate canonical WNT/β-catenin signaling. Bangle extract-treatment of hfNSCs not only promoted neuronal differentiation, but also accelerated neurite outgrowth from immature neurons. Furthermore, Bangle extracts induced expression of neurogenic genes and WNT signaling-target genes, and facilitated the accumulation of β-catenin in nuclei of hfNSC. Interestingly, altered histone modifications were also observed in Bangle-treated hfNSCs. Together, these findings demonstrate that Bangle contributes to hfNSC neurogenesis by WNT pathway and epigenetic regulation.

## 1. Introduction

Multipotent neural stem cells (NSCs) have the potential to both self-renew and differentiate into neurons, astrocytes, and oligodendrocytes in the mammalian central nervous system (CNS) [1,2,3]. This is tightly regulated by cell-extrinsic factors, such as cytokines and morphogens, and epigenetic modifications including DNA methylation and histone modification [4,5]. Recent reports indicate that NSCs derived from the cortex of the fetal mammalian brain share distinct features across species [6,7,8]. In rodents, cortical NSCs of fetal brain can differentiate into excitatory neurons, but not inhibitory neurons, while human NSCs can differentiate into both neuronal types. In addition, primate basal radial glia (bRG) can proliferate in the outer subventricular zone (oSVZ), resulting in an increased number of neurons, ultimately leading to the establishment of the large primate neocortex. Thus, human NSCs are more desirable for therapeutic agent screening and the investigation of diets that have a beneficial role in neurodegenerative diseases.

Bangle, or Indonesian ginger (*Zingiber purpureum*) is a tropical ginger widely distributed in Southeast Asia. Previous studies using the rhizomes of *Zingiber cassumunar* (a *Z. purpureum* synonym) have reported that the isolated rhizome compounds exhibit a number of biological activities such as anti-inflammatory and analgesic activities [9,10,11,12,13], and cytotoxic activity [14,15,16,17]. Moreover, two phenylbutenoid dimers, *cis*- and *trans*-3-(3′4′-dimethoxyphenyl)-4-[(*E*)-3″,4″-dimethoxystyryl] cyclohex-1-ene, were identified as a Bangle extract called “*cis/trans*-Banglene” (Figure S2 [18]). Recent studies have shown that these dimers extracted from Bangle can not only induce neuritogenesis in PC12 cells, but they also exhibit neurite elongation and protective activity against cell death caused by serum-deprivation in primary cultured mouse cortical neurons [18,19]. Moreover, dietary intake of *cis/trans*-Banglene enhanced hippocampal neurogenesis in olfactory bulbectomized (OBX) mice, which led to a specific set of changes in social behavior, cognitive function and activity [18]. On the other hand, a diet containing these compounds improved spatial learning and memory deficits in the senescence-accelerated prone (SAMP8) mouse, an animal model of spontaneous overproduction of amyloid precursor protein (APP) and oxidative damage, suggesting that Bangle extract has neurotrophin-like activity and is beneficial for the prevention of age-related progression of cognitive impairment [19]. However, the underlying molecular mechanisms of Bangle extract-induced neurogenesis are still to be determined. Additionally, it has not yet been examined whether Bangle displays neurotrophin-like activity on human NSCs.

In this study, we demonstrate that Bangle extract exhibits neurogenic properties on human fetal NSCs (hfNSCs) and facilitates neurite outgrowth in immature neurons differentiated from hfNSCs. Moreover, Bangle not only activates the WNT signaling pathway, but also alters histone modifications in hfNSCs. Our findings suggest that Bangle may be an attractive candidate for reducing the symptoms of neurological disorders and assisting with neurorehabilitation.

## 2. Results

### 2.1. Bangle (Zingiber purpureum) Extract Promotes Neuronal Differentiation of hfNSCs and Enhanced Neurite Outgrowth of Immature Neurons

**Figure 1:**
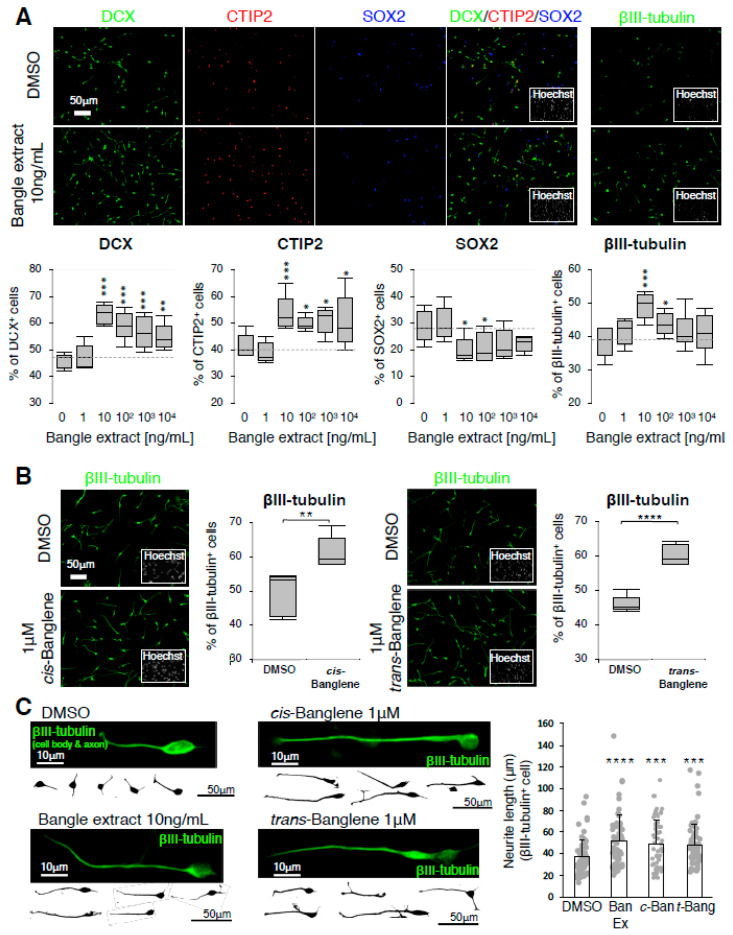
Bangle extract promotes neuronal differentiation of human fetal neural stem cells (hfNSCs) and enhances neurite outgrowth of premature neurons. (**A**,**B**) Cells treated with a series of concentrations of Bangle extract and 1 µM *cis/trans*-Banglene were stained with several antibodies 7 days after induction of differentiation. The graphs indicate the percentage of total cells that were DCX-, CTIP2-, SOX2-, or βIII-tubulin-positive. The values shown are the means ± SD. (*N* = 5, ANOVA, * *p* < 0.05, ** *p* < 0.01, *** *p* < 0.005, **** *p* < 0.001). (**C**) Representative images of single βIII tubulin-positive neurites 4 days after induction of neuronal differentiation. Neurite length of immature neurons differentiated from hfNSCs is shown as mean ± SEM. (*N* = 60, *** *p* < 0.005, **** *p* < 0.001). Grey dots represent the value from individual cells.

First, to examine whether Bangle extract affected neuronal differentiation of hfNSCs, we treated hfNSCs with Bangle extract dissolved in dimethyl sulfoxide (DMSO), and this led to spontaneous neuronal differentiation (Figure S1). Seven days post-differentiation we observed that compared to DMSO-treated control cells, Bangle extract-treated cells exhibited an increased percentage of cells that expressed doublecortin (DCX, immature neuron), CTIP2 (excitatory neuron) and βIII-tubulin (early neuron) and a reduction in the percentage of SOX2 (NSC)-positive cells (Figure 1A). Given that 10 ng/mL of Bangle extract was the most effective concentration for the promotion of hfNSC differentiation, we used it in all subsequent assays. We then investigated the effect of *cis* and *trans* isomers of Banglene, the reported active compounds of Bangle [20], on neuronal differentiation of hfNSCs and found that both isoforms also increased the percentage of βIII-tubulin- (Figure 1B), DCX- and CTIP2-positive cells, while reducing that of SOX2-positive cells (Figure S2). Together, these results indicate that both Bangle extract and *cis/trans*-Banglene have the ability to promote neuronal differentiation of hfNSCs. Next, to test whether Bangle extract influenced neuronal growth, we evaluated neurite outgrowth of immature neurons differentiated from hfNSCs 4 days after treatment with Bangle extract and *cis/trans*-Banglene. The hfNSC neurites stained by βIII-tubulin in each immature neuron were significantly elongated compared to control neurons (Figure 1C). However, at 7 days post-treatment, there was no difference in neurite length between control and Bangle extract-treated neurons (Figure S3). These data indicated that Bangle extract might be related to the initial phase of neurite outgrowth. Notably, we also observed no difference in the uptake of PI and EdU between the DMSO-, Bangle extract-, or *cis/trans*-Banglene-treated hfNSCs (Figure S4A–C), which suggests that these treatments do not affect cell death and proliferation of hfNSCs. Taken together, these data indicate that Bangle extract and *cis/trans*-Banglene promote neuronal differentiation and accelerate neurite outgrowth of immature neurons during early periods of neuronal growth.

### 2.2. Bangle Extract Affects the Expression of Genes Related to Neurogenesis and WNT Pathway

**Figure 2:**
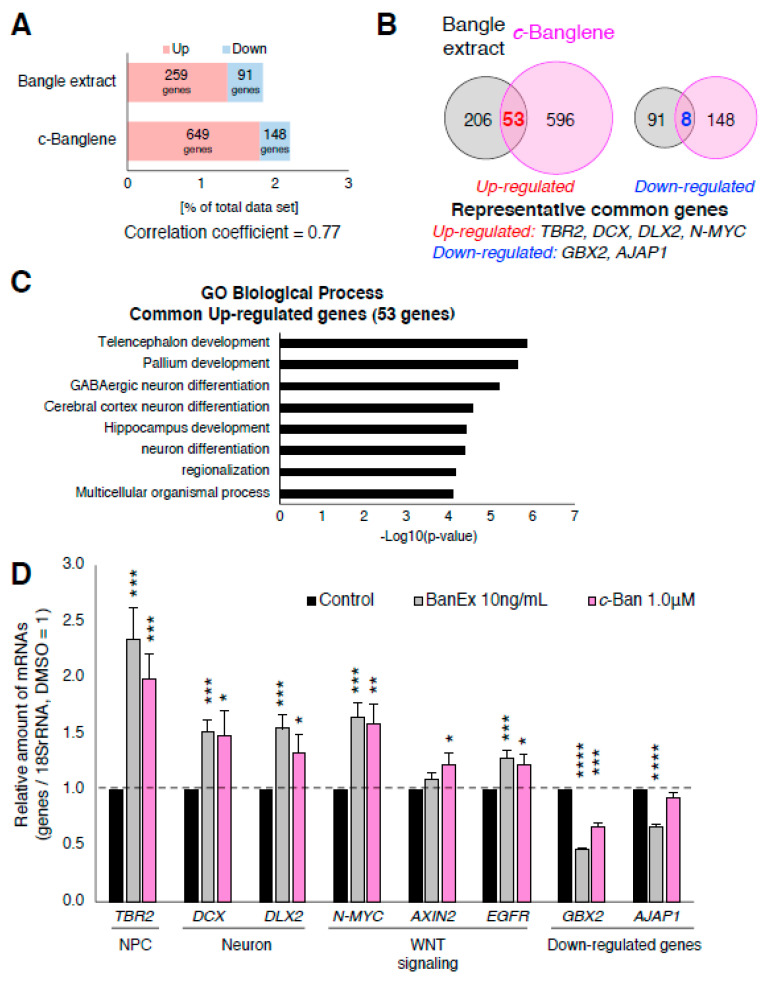
Bangle extract and Banglene affect the expression of genes related to neurogenesis and WNT pathway. (**A**) Microarray analysis of hfNSCs 2 days after differentiation. The graph shows percentage of up- and down-regulated genes in Bangle extract (N=3) and *c*-Banglene (N=2) treated cells as compared to untreated cells (upregulated gene > 1.3-fold; downregulated genes < 0.7-fold; *P* < 0.05). (**B**) Venn diagram of unique and shared mRNAs in Bangle extract and *c*-Banglene-treated cells. (**C**) Gene ontology (GO) analysis of the commonly upregulated genes. Analysis was performed by PANTHER (http://pantherdb.org/). (**D**) Quantitative RT-PCR analysis of the cells at 2 days after the induction of differentiation. Data represent mean ± SEM, when compared to control cells. (*N* = 6, *t*-test, * *p* < 0.05, ** *p* < 0.01, *** *p* < 0.005, **** *p* < 0.001).

To investigate the mechanisms by which Bangle extract and *cis*-Banglene (*c*-Banglene) promoted neurogenesis of hfNSCs, we performed genome-wide gene expression profiling of control and extract-treated hfNSCs via microarray analysis. As shown in Figure 2A, 259 and 649 genes were significantly upregulated in Bangle extract- and *c*-Banglene-treated hfNSCs, respectively, with 53 of those genes commonly upregulated by the treatments after 2 days (Figure 2B and Tables S1 and S2). On the other hand, 91 and 148 genes in Bangle extract- and *c*-Banglene-treated hfNSCs, respectively, were identified as significantly downregulated genes (Figure 2A,B). We estimated the Pearson’s correlation coefficient between the microarray data sets of relative gene expression profiles in treatments with Bangle extract and *c*-Banglene, and found a correlation coefficient of 0.77 (*p* < 0.001), indicating a positive correlation between these genetic expression profiles (Figure 2A). Gene ontological analysis revealed that the most commonly upregulated genes included genes involved with forebrain development and neuronal differentiation, such as Eomesodermin (*EOMES*, or *T-box brain protein 2* (*TBR2*)), *DCX*, and *Distal-less homeobox 2* (*DLX2*) (Table S1, Figure 2B,C). Notably, we also observed upregulation of *N-MYC,* a target gene of the canonical WNT/β-catenin signaling pathway, after Bangle extract and *c*-Banglene treatment (Table S1, Figure 2B). Real time-PCR analysis also revealed significant upregulation of genes related to neuronal differentiation (*TBR2*, *DCX*, and *DLX2*) and downstream target genes of WNT signaling (*N-MYC*, *EGFR* [21]) (Figure 2D). The significant upregulation of *AXIN2*, the expression of which is dependent on activation of the WNT signaling pathway [22], was only observed in *c*-Banglene treated-cells in qRT-PCR analysis (Figure 2D). Given that activation of WNT signaling in neural stem cells promotes neuronal differentiation in the developing mouse neocortex [23], our gene expression analyses led us to hypothesize that the WNT/β-catenin signaling pathway participates in the promotion of neurogenesis by Bangle.

The most common downregulated genes in Bangle extract- and *c*-Banglene-treated hfNSCs included *gastrulation brain homeobox 2* (*GBX2*), a negative regulator of forebrain development [24], and *adherens junctions associated protein 1* (*AJAP1*), a negative regulator of Wnt signaling [25]. The downregulation of these genes was confirmed by qRT-PCR, although the statistically significant reduction of *AJAP1* expression in *c*-Banglene was not observed in this assay (Figure 2D). These results also implied that Bangle extract and *c*-Banglene treatments indirectly induce the activation of WNT signaling and the neurogenesis of hfNSCs through the downregulation of these genes.

### 2.3. Bangle Extract Activates the Canonical WNT/β-Catenin Signaling Pathway in hfNSCs

**Figure 3:**
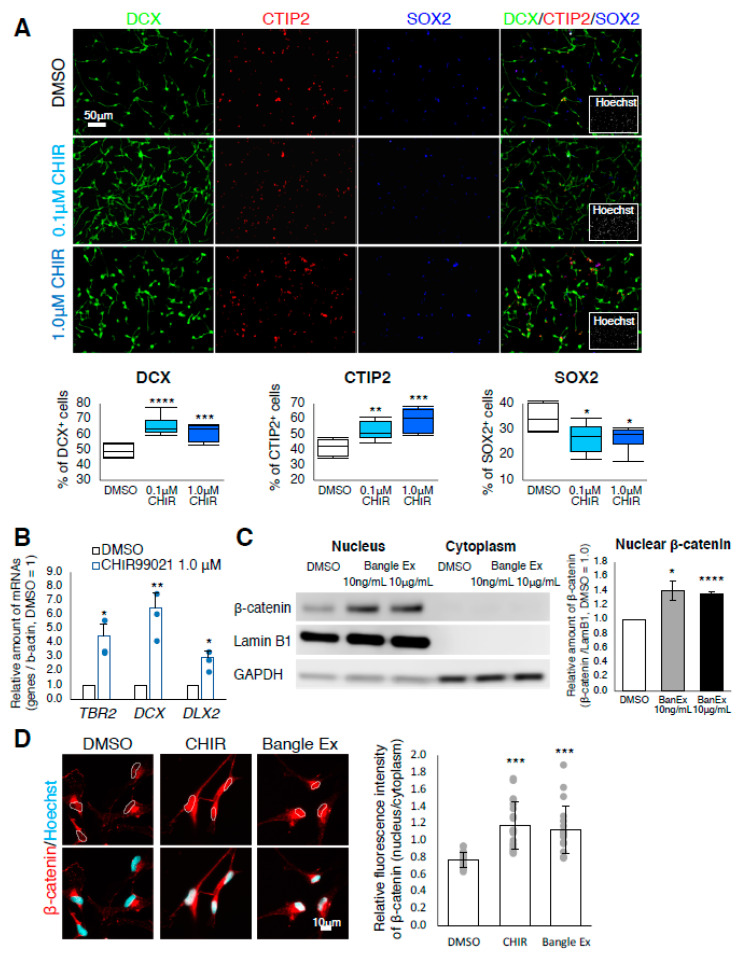
Treatment with the WNT signaling activator CHIR99021 promotes neuronal differentiation of hfNSCs. (**A**) Cells treated with or without CHIR99021 were stained with several antibodies 7 days after induction of differentiation. Scale bar: 50 µm. The graphs indicate the percentage of total cells that were DCX-, CTIP2-, or SOX2-positive. The values shown are the means ± SD. (*N* = 6, *t*-test, * *p* < 0.05, ** *p* < 0.01, *** *p* < 0.005, **** *p* < 0.001). (**B**) Quantitative RT-PCR analysis of the cells 2 days after the induction of differentiation. Data represent mean ± SEM, when compared with control cells. (*N* = 3, *t*-test, * *p* < 0.05, ** *p* < 0.01). Blue dots represent the value from individual experiments. (**C**) Western blot analysis of nuclear and cytoplasmic β-catenin expression in 10 ng/mL and 10 µg/mL Bangle extract-treated hfNSCs. LaminB1 and GAPDH were used as nuclear and cytoplasmic fraction markers, respectively. In the graphs, the values of nuclear β-catenin derived from ImageStudio Digits were normalized by LaminB1. The relative amounts are the means ± SEM compared with those for DMSO treatment. (*N* = 3, *t*-test, * *p* < 0.05, **** *p* < 0.001). (**D**) The cells treated with 10 µM CHIR99021 and 10 ng/mL Bangle extract for 2 days were stained with antibodies against β-catenin (red) and Hoechst (nucleus: cyan). Scale bar: 10 µm. Quantification of the ratio of fluorescence intensity of nuclear β-catenin to that of cytoplasm. The values represent the means ± SD. (*N* = 20, *t*-test, *** *p* < 0.005). Grey dots represent the value from individual cells.

To test the hypothesis that activation of the WNT/β-catenin pathway induces neurogenesis of hfNSCs, we treated hfNSCs with CHIR99021, which activates the WNT/β-catenin signaling pathway by blocking the formation of the Axin-GSK3β complex. As shown in Figure 3A, the percentage of DCX-positive and CTIP2-positive neurons was significantly increased in CHIR99021-treated hfNSCs compared to control cells, whereas the percentage of SOX2-positive NSCs was decreased (Figure 3A), indicating that the activation of canonical WNT/β-catenin signaling is sufficient to induce neuronal differentiation. We further examined the expression of genes upregulated by Bangle extract and *c*-Banglene in control and CHIR99021-treated hfNSCs. Marked induction of TBR2, DCX and DLX2 in CHIR99021-treated hfNSCs was observed by qRT-PCR analysis (Figure 3B), suggesting that the induction of neuronal differentiation by activation of the canonical WNT/βcatenin signaling pathway in hfNSCs is caused by the upregulation of these genes.

To directly test whether Bangle extract activates the WNT/β-catenin pathway, we evaluated the amount of nuclear β-catenin via Western blot in control, CHIR99021-treated and Bangle extract-treated hfNSCs. We observed that the amount of β-catenin was increased in the nucleus of Bangle extract-treated hfNSCs as well as in CHIR99021 treated-cells, a positive control (Figure S5A). Quantification by signal intensity in Western blot analysis revealed the significant enrichment of β-catenin in the nucleus with treatment of 10 ng/mL and high-concentration (10 µg/mL) of Bangle extract (Figure 3C), suggesting that 10 ng/mL Bangle extract treatment is sufficient to activate Wnt/β-catenin signaling. The levels of cytoplasmic β-catenin were too low compared with that of nucleus in both control and Bangle extract-treated cells to exactly measure these signal intensities (Figure 3C and Figure S5B). Immunocytochemistry also revealed accumulation of β-catenin in the nuclei of CHIR99021-treated and Bangle extract-treated hfNSCs (Figure 3D). Collectively, these data indicate that Bangle extract directly activates the canonical WNT/βcatenin signaling pathway in hfNSCs.

### 2.4. Canonical WNT/β-Catenin Signaling Is Responsible for the Promotion of Neuronal Differentiation in Response to Bangle Extract Treatment

**Figure 4:**
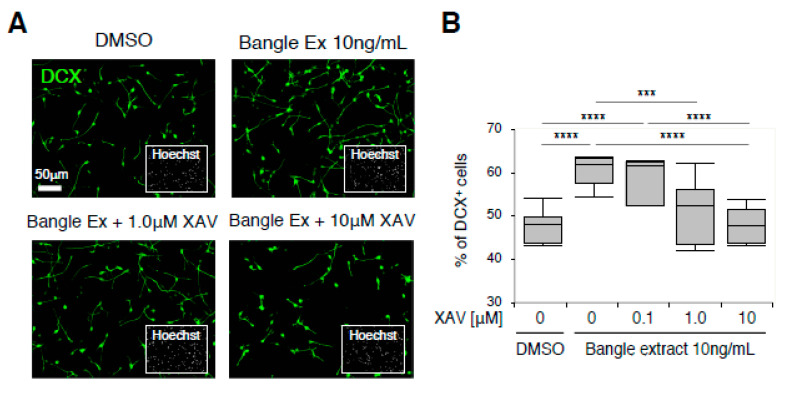
WNT signaling is responsible for the promotion of neuronal differentiation in response to Bangle extract treatment. (**A**) Cells treated with 10 ng/mL Bangle extract and the WNT signaling inhibitor XAV-939 were stained with antibodies against DCX (green) 7 days after induction of differentiation. (**B**) The graphs indicate the percentage of total cells that were DCX-positive. The values shown are the means ± SD. (*N* = 6, ANOVA, *** *p* < 0.005, **** *p* < 0.001).

We further examined whether pharmaceutical inhibition of canonical WNT/β-catenin signaling blocked the neurogenic activity of Bangle extract on hfNSCs. XAV-939, a WNT signaling inhibitor that induces Axin2-dependent β-catenin-degradation, was combined with Bangle extract and hfNSCs, and these hfNSCs underwent the induction of neuronal differentiation. Although the ratio of DCX-positive cells was increased by Bangle extract treatment alone, this increase was attenuated by the treatment with XAV-939 in a concentration dependent manner (Figure 4A,B). Further, 10 µM of XAV-939 completely blocked the neurogenic effect of Bangle extract treatment in hfNSCs (Figure 4B). The administration of IWR-1-endo, an inhibitor to stabilize β-catenin degradation complex as well as XAV-939, showed similar effects to XAV-939 on neuronal differentiation of hfNSCs treated with Bangle extract (data not shown [26]). Taken together, these results demonstrated that the activation of the canonical WNT/β-catenin signaling pathway is indispensable for the neurogenic effect of Bangle extract on hfNSCs.

### 2.5. Bangle Extract Affects Histone Modifications during Neuronal Differentiation

**Figure 5:**
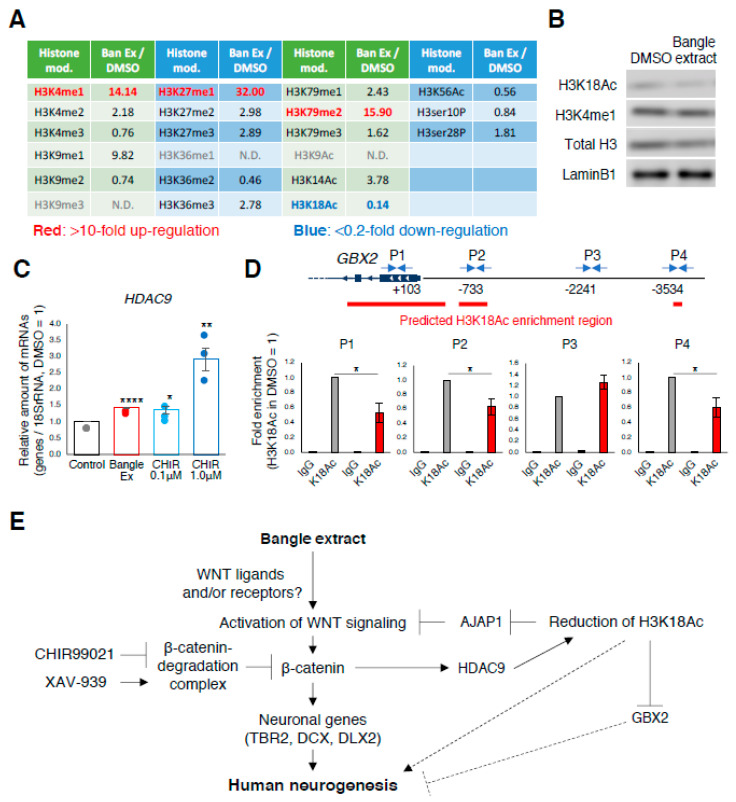
Bangle extract affects histone modifications during neuronal differentiation. (**A**) The cells were treated with 10 ng/mL Bangle extract for 2 days during the induction of neuronal differentiation. Equal amounts of nuclear protein were added among samples in the experiment. Each histone H3 modification is presented as a ratio normalized to DMSO-treated controls. (**B**) Western blot analysis of nuclear H3K18Ac expression in Bangle extract-treated (10 ng/mL) hfNSCs. (**C**) Quantitative RT-PCR analysis of the cells 2 days after the induction of differentiation. Data represent mean ± SEM, when compared to control cells. (*N* = 3, *t*-test, * *p* < 0.05, ** *p* < 0.01, **** *p* < 0.001). Colored dots represent the value from individual experiments. (**D**) ChIP assays for hfNSC at 2 days after induction of differentiation were performed using anti-H3K18Ac antibody, and control IgG, followed by real-time PCR with the primer sets in the proximity of *GBX2* gene. The relative amounts are the means ± SEM compared with those for H3K18Ac in DMSO treatment. (*N* = 3, *t*-test, * *p* < 0.05). (**E**) Summary of the promotion of human neurogenesis by treatment of Bangle extract and its active ingredient.

The microarray analysis revealed the altered expression of many genes after treatment with Bangle extract and its active ingredients, implying that Bangle extract also influenced genome-wide epigenetic modifications, including histone proteins. Therefore, we assessed global changes in histone H3 methylation, acetylation, and phosphorylation after Bangle extract treatment in hfNSCs by using a modified ELISA method (Figure 5A). We found that the treatment of Bangle extract altered the methylation or acetylation status of lysine residues on histone H3, including a marked reduction in the amount of acetylated histone H3 lysine 18 (H3K18Ac), a histone deacetylase-9 (HDAC9) target. We performed Western blotting and confirmed the degradation of H3K18Ac in Bangle extract-treated hfNSCs (Figure 5B). However, the remarkable upregulation of H3K4me1 was not detected by Western blotting in spite of global analysis by ELISA (Figure 5B). We found that with the decrease of acetylated H3K18, the expression of HDAC9 was increased in treatment with Bangle extract and CHIR99021 (Figure 5C). Given that H3K18Ac is a general marker for active transcription [27], it was hypothesized that the decrease in H3K18Ac enrichment occurred at the downregulated genes by Bangle extract-treated hfNSCs. To test this hypothesis, we performed chromatin immunoprecipitation (ChIP)-qPCR analysis using specific primers to detect regulatory regions of *GBX2* and *AJAP1* gene [28]. As shown in Figure 5D, we confirmed the depression of H3K18Ac enrichment on *GBX2* in Bangle extract-treated cells compared with that of DMSO-treated cells, as well as *AJAP1* gene (Figure S6). Altogether, these results suggest that Bangle extract causes epigenetic alterations, including H3K18 deacetylation, which may contribute to both hfNSC neuronal differentiation and the neurite outgrowth of immature neurons differentiated from hfNSCs.

## 3. Discussion

It is well-known that activation of the canonical WNT/β-catenin signaling pathway induces neuronal differentiation of NSCs both in vitro and in vivo [23]. The canonical WNT/β-catenin signaling pathway is activated by the binding of the WNT ligands to cell membrane receptors, thereby triggering downstream events that culminate in the accumulation of β-catenin in the cytoplasm and its translocation into the nucleus [29,30,31]. The interaction of β-catenin with transcription factors of the TCF/LEF family in the nucleus modifies the gene expression of crucial genes, thus leading to changes in key cellular pathways, such as proliferation, migration and cell fate [32]. Moreover, canonical WNT/β-catenin signaling induces the end of self-renewal by mouse cortical neuronal progenitor cells (NPCs) and directs their neuronal differentiation in a later stage of development [23]. Here, we provide, to the best of our knowledge, the first evidence that Bangle extract also activates the canonical WNT/β-catenin signaling pathway and induces neurogenesis in human NSCs.

Microarray analysis revealed upregulation of TBR2, DCX, and DLX2, among other genes, after Bangle extract and *c*-Banglene treatment. Previous studies have suggested that WNT/β-catenin signaling is involved in regulating the expression of TBR2, which is a marker of intermediate progenitor cells and is essential for neurogenesis [33,34], and DCX in NSCs of humans and mice [35,36], and it regulates DLX2 expression in the fetal mouse brain [37,38]. DLX2 expression in NSCs is known to be sufficient for differentiation into GABAergic neurons [39]. Therefore, our study suggests that Bangle extracts induce neurogenesis via activating WNT/β-catenin signaling in human fetal neural stem cells. We also found that HDAC9 was increased in Bangle extract-treated and CHIR99021-treated cells (Figure 5C), suggesting that the expression of HDAC9 is regulated by WNT signaling, and that histone modification in these cells might change after treatment with Bangle extracts. Indeed, ELISA analysis revealed global changes in histone modification, including H3K18Ac controlled by HDAC9 in Bangle extract-treated hfNSCs (Figure 5A). It is well-known that histone modification plays an important role in neuronal differentiation and neurite growth in the mammalian fetal brain [4]. Future studies should focus on how these epigenetic changes regulate neurogenesis during human fetal development.

A recent study reported that AJAP1 functions as a negative regulator of WNT signaling through preventing nuclear translocation of β-catenin by anchoring to cell membrane in breast cancer cell [25]. Here, we observed the reduction of *AJAP1* gene in Bangle extract-treated cells (Figure 2D). This reduction may impact on the nuclear translocation of β-catenin and contribute to the activation of WNT signaling. Meanwhile, as shown in Figure 2D, slight but significant upregulation of *AXIN2*, which is a factor that negatively controls WNT signaling, was observed in *c*-Banglene-treated hfNSCs. This increase might attenuate the activation of WNT signaling by this treatment, and results in the difference in gene expression profiles between Bangle extract and *c*-Banglene.

A previous study showed that Bangle-derived compounds, as well as fluoxetine, improved impaired hippocampal neurogenesis in OBX mice. Notably, administration of fluoxetine increases Wnt3a protein in the dentate gyrus of the hippocampus [40] and markedly enhances hippocampal neurogenesis [41]. Accordingly, we suggest that the improvement in neurogenesis by Bangle administration in the OBX-mouse hippocampus is partially dependent on WNT signaling activation.

In this study, we demonstrated that Bangle extract induces the accumulation of β-catenin in the nucleus of hfNSCs and facilitates the neurogenesis of hfNSCs (Figure 3C). However, Bangle extract was not as effective as CHIR99021 in inducing expression of neuronal genes in hfNSCs. This result suggests that unlike CHIR99021, Bangle extract may not directly prevent formation of the Axin-GSK3β complex. Additionally, we have previously shown that activation of lysine specific demethylase 1 (LSD1) via influx of flavin adenine dinucleotide (FAD), which acts as a coenzyme of LSD1, promotes neurogenesis of hfNSCs [42], suggesting that activation of pathways other than canonical WNT/β-catenin signaling may contribute to Bangle extract-promotion of neurogenesis in hfNSCs. However, the neurogenic effect of Bangle extract on hfNSCs was completely counteracted by treatment with XAV939, an inhibitor of Tankyrase (TNKS) enzyme. By inhibiting TNKS activity, XAV939 increases the protein levels of the axin-GSK3β complex and promotes the degradation of β-catenin, thereby preventing the canonical WNT pathway. Treatment with XAV939 alone did not impair spontaneous neuronal differentiation of hfNSCs, as shown by comparison with the reduction of spontaneous neuronal differential after withdrawal of mitogen (data not shown), which suggests that activation of the canonical WNT signal pathway via TNKS activity is not necessary for the neurogenesis of cultured hfNSCs. Therefore, our findings prompt us to hypothesize that Bangle extract targets more upstream molecules of the WNT signaling pathway, such as the ligands and receptors, including Frizzled. More studies are required to clarify this hypothesis.

Many epigenetic changes are observed in response to the up or downregulation of epigenetic modifiers during neuronal differentiation of mouse NSCs, and these epigenetic modifications play a crucial role in neuronal differentiation and also in neuronal growth [4]. One epigenetic modifier we found in our study is HDAC9, which is a class IIa histone deacetylase that targets lysine residues 9, 14, and 18 on histone H3 [43,44]. HDAC9 is known to play an important role in the neuronal development of mice [45]. We found that, accompanied by the upregulation of HDAC9, deacetylation of H3K18 in hfNSCs was facilitated by treatment with Bangle extract. We also observed that the treatment with CHIR99021 induced HDAC9 expression (Figure 5C), suggesting that upregulation of HDAC9 caused by Bangle treatment in hfNSCs is dependent on activation of the canonical WNT signaling pathway. We further found decreased enrichment of H3K18 acetylation at the proximity regions of *GBX2* and *AJAP1* in Bangle extract-treated hfNSCs. Given that GBX2 expression disturbs forebrain development in mouse [24], our findings suggest that Bangle extract also contributes to neurogenesis through preventing H3K18Ac enrichment on regulatory regions of these genes. In addition, it has been reported that HDAC9 mediates dendrite growth in post-mitotic neurons derived from the developing mouse brain [46,47], and that deacetylation activity of HDAC9 is required to control the length of dendrites [47]. Therefore, H3K18 deacetylation induced by Bangle extract may promote neurite outgrowth of premature neurons differentiated from hfNSCs. Moreover, the promoting effect of Bangle extract on neurites of PC12 cells that was indicated in a previous study, might rely on HDAC9 upregulation [18].

In this study, there was clear variation in the gene expression profiles of Bangle extract and *c*-Banglene, and we also found different expression levels of genes, such as *AXIN2, HDAC9* and *AJAP1*, in the qRT-PCR analysis (Figure 2D, Figure 5C and Figure S7). Upregulation of *AXIN2* was only observed in *c*-Banglene-treated hfNSCs, and the expression of *AJAP1* was significantly downregulated in Bangle extract-treated hfNSCs, but not *c*-Banglene-treated cells. These results suggested that the degree of WNT signaling activations was probably different for the Bangle extract and *c*-Banglene. Moreover, we did not observe a significant increase in HDAC9 expression in *c*-Banglene treated hfNSCs in contrast to that of Bangle extract (Figure S7), suggesting that the level of H3K18Ac enrichment on each gene is also different between each hfNSC. Given all this, it is hypothesized that the variable gene expression profiles among Bangle-extract and *c*-Banglene may be caused by the differences in *AXIN2*, *AJAP1* and *HDAC9* expression.

Bangle extract may also affect other epigenetic modifications, such as DNA methylation and hydroxymethylation. Indeed, our microarray analysis has revealed the increased expression of *GADD45G*, a member of a family of proteins (Growth Arrest and DNA Damage response) implicated in DNA repair and active DNA demethylation [48,49], in both Bangle extract and *c*-Banglene-treated hfNSCs (Table S1), suggesting that Bangle extract impacts on DNA methylation through the regulation of *GADD45G* expression. In the future, further investigation of the effect of Bangle extract on DNA methylation in hfNSCs is required.

Although we demonstrated here that Bangle extract activates WNT signaling pathway in hfNSCs, it is still unclear whether the extract also effects the NSCs of fetal or adult human brain in vivo. Cerebral organoids, which are novel three-dimensional (3D) cerebral models derived from human embryonic or induced pluripotent stem cells, mimic the human prenatal development in vitro [50,51,52]. The organoids more closely resemble the natural environment of cells, cell-matrix interactions, and complex transport systems of nutrients [50,51,52]. Therefore, studies using the organoids could give extremely valuable insight into the effects of Bangle extract on the human brain in the future.

In this study, we have shown that Bangle extract induces neurogenesis of human NSCs via the activation of the canonical WNT signaling pathway and histone modifications. It has been reported that activation of WNT signaling rescues memory loss and improves synaptic dysfunction in mouse models of Alzheimer’s disease, such as the SMAP8 mouse and the APP/PS1-transgenic mouse. A recent study suggested that ingestion of Bangle extract tablets was safe for at least one month in human clinical studies [53]. Therefore, dietary intake of Bangle may be effective not only for enhancing adult neurogenesis, but also for combating Alzheimer’s disease. In addition, accumulated evidence has demonstrated that neurorehabilitation can help to repair central and peripheral nervous impairment caused by stroke, spinal cord injury, and other neurological diseases, including Alzheimer’s disease [54,55,56]. It is expected that the combination of dietary Bangle intake and neurorehabilitation will be an effective therapy for the functional maintenance and recovery of the central and peripheral nervous system in patients with various neurological disorders.

## 4. Material and Methods

### 4.1. Human Fetal Neural Stem Cell Culture, In Vitro Differentiation and Immunostaining

All human cell and tissue experiments were approved by the Human Experiment Committee and Ethics Committee of the National Institute of Advanced Industrial Science and Technology (approval No. 2013-656C, 10 September 2015). hfNSCs were purchased from PhoenixSongs Biologicals, Inc. (PSB, Branford, CT, USA) (Cat# 23001-003, Donor Lot CxB-3). In preparation for establishing hfNSC lines, informed consent was obtained from the donor, or donor’s next of kin by the PSB company (https://phoenixsongsbio.com/). The cell line is derived from the human cerebral cortex of a male fetus at embryonic week 14. Culture methods for hfNSCs have been described previously [42,57], and the neural aspect of this cell line confirmed. In brief, hfNSCs were cultured in N2-supplemented Dulbecco’s modified Eagle’s medium with F12 (DMEM/F12, GIBCO, Waltham, MA, USA) containing a 0.1% B27 supplement (GIBCO), 10 ng/mL human basic fibroblast growth factor (FGF; R&D Systems Inc., Minneapolis, MN, USA), and 20 ng/mL human epidermal growth factor (EGF; PeproTech, Inc., Rocky Hill, NJ) on culture dishes that had been precoated with poly-l-ornithine (Sigma-Aldrich, St. Louis, MO, USA) and laminin (Corning, Corning, NY, USA). A maximum of 30 cell-passages were used. For neuronal differentiation, hfNSCs were placed into neurobasal medium (GIBCO) containing 2% B27 supplement (GIBCO) and 0.5 mM l-glutamine (Nacalai Tesque, Inc., Kyoto, Japan).

For immunostaining, cells were washed with phosphate-buffered saline (PBS), fixed in 4% paraformaldehyde in PBS, and stained with appropriate antibodies (Table S3). Before fixation, 2 µg/mL propidium iodide (PI) or 10 µM 5-ethynyl-2′-deoxyuridine (EdU) was added to the culture medium for 10 min or 120 min, respectively, at 37 °C. Nuclei were stained after fixation using Hoechst 33342 (Dojindo Laboratories, Kumamoto, Japan). Stained cells were visualized with a fluorescence microscope (BX53, Olympus, Tokyo, Japan).

### 4.2. Bangle Extract and cis/trans-BANGLENE

Bangle extract powder (Hosoda SHC Co., Fukui, Japan) contained *trans*- and *cis*-3-(3′,4′-dimethoxyphenyl)-4-[(*E*)-3″,4″-dimethoxystyryl]cyclohex-1-ene (phenylbutenoid dimers; 5.0%) and was composed of 20.2% Bangle extract, 8.5% emulsifier, and 71.3% dextrin [58].

### 4.3. Measurement of Neurite Length

The hfNSCs were plated in 35 mm culture dishes at a density of 1 × 10^5^ cells/dish. After neuronal differentiation with 10 ng/mL Bangle extract and 1 µM *cis/trans*-Banglene treatments, the cells were fixed and immunostained by anti-βIII tubulin antibody. The length of the longest neurite in each βIII tubulin-positive cell was assessed by using Image J v. 1.49. Experiments were performed three times independently and 20 cells were counted in each experiment.

### 4.4. Analysis of Nuclear Translocation of β-Catenin by Immunocytochemistry

The hfNSCs were plated in 35 mm culture dishes at a density of 5 × 10^4^ cells/dish overnight. One day after seeding, the medium was replaced by fresh undifferentiation medium including 10 ng/mL Bangle extraction for 2 days. After fixation, the cells were stained by anti-β-catenin antibody and Hoechst and observed by a fluorescence microscope (BX53, Olympus). Fluorescence intensities of β-catenin in nucleus and cytoplasm were measured by using Image J. That value of nuclear β-catenin was normalized to that of cytoplasm. Experiments were performed two times independently and 10 cells were counted in each experiment.

### 4.5. Quantitative Reverse-Transcription Polymerase Chain Reaction

Total RNA was isolated from hfNSCs that had been treated with either Bangle extract, *c*-Banglene, or CHIR99021. Reverse transcription was performed with SuperScript VILO reverse transcriptase (Invitrogen) and real-time PCR was performed with KAPA SYBR Fast (KAPA Biosystems, Inc., Wilmington, MA, USA). The primers used are described in Table S4.

### 4.6. Nuclear Extract and Western Blot Analysis

Two days after treatment with Bangle extract or CHIR99021, the hfNSCs were first washed with cold PBS, and then hypotonic buffer (20 mM Tris-HCl pH 7.4, 10 mM NaCl, 1.5 mM MgCl_2_) was added. The nuclear extract was retrieved by centrifugation (800× *g*). Pellet samples (nuclei) and supernatant (almost pure cytoplasm), for immunoblotting, were prepared as follows. Pellets were lysed with lysis buffer (50 mM Tris-HCl pH 7.4, 150 mM NaCl, 1% Triton X-100) that included a protease inhibitor cocktail (Nacalai Tesque, Inc.) and a phosphatase inhibitor cocktail (Nacalai Tesque, Inc.) for 30 min on ice. Then, sample buffer (Nacalai Tesque, Inc.) was added to the pellet lysed samples and supernatant samples, and these samples were incubated for 5 min at 95 °C. Aliquots of total cell lysate were separated by 5–20% SuperSep (Wako Pure Chemical Industries, Ltd., Osaka, Japan) and transferred onto polyvinylidene fluoride membranes (EMD Millipore, Billerica, MA). After blocking with BlockingOne (Nacalai Tesque, Inc.) containing 0.1% Tween 20, the membranes were incubated with appropriate primary antibodies (Table S3). After washing with TBST (Tris-buffered saline, 0.1% Tween 20), the membranes were then incubated with the appropriate HRP-conjugated secondary antibodies (Abcam), washed, and developed with ECL Prime reagents (GE Healthcare Bio-Sciences, Pittsburgh, PA, USA).

### 4.7. Microarray and Gene Ontology Analysis

Total RNA isolated from hfNSCs 2 days after neuronal differentiation and treatment with Bangle extract or *c*-Banglene were hybridized to whole-human gene expression microarrays (SurePrint G3 Human Gene Expression 8x60K v. 3, Agilent) according to the manufacturer’s instructions. The results from three independent experiments were then filtered using significance-threshold *p*-values from unpaired two-tailed Student’s *t*-tests for single comparisons (*p* < 0.05). Data was then uploaded to the Gene Expression Omnibus (GEO accession No: GSE129415.). With regard to the expression level of *DCX*, a gene that was increased more than 1.3-fold in comparison with DMSO-treated hfNSCs was defined as a significantly upregulated gene in Bangle extract- and *c*-Banglene-treated hfNSCs. Accordingly, a gene that was decreased less than 0.7-fold in comparison with DMSO-treated hfNSCs was defined as a significantly downregulated gene. Gene ontology analysis (GO, biological process) for increased gene expression under both treatment conditions (>1.3-fold) was performed using PANTHER (http://www.pantherdb.org/) consortium databases [59]. The Pearson’s correlation coefficient was estimated between the microarray data sets of relative gene expression profiles in treatments with Bangle extract and *c*-Banglene.

### 4.8. Histone H3 Modification Analysis by ELISA

Histone H3 modification analyses were performed using an H3 modification multiplex array (EpiQuik Histone H3 Modification Multiplex Assay Kit, #P-3100-96, Epigentek). hfNSCs were treated with 10 ng/mL Bangle extract during induction of neuronal differentiation. Two days after induction, the cells were lysed and their nuclei extracted. Experiments were conducted using equal amounts of nuclear protein (100 ng/well) per sample. H3 modifications (relative to protein amounts) were calculated for each sample according to the manufacturer’s instructions, and were presented as a ratio normalized to a DMSO-treated control.

### 4.9. Chromatin-Immunoprecipitation Assay

Chromatin immunoprecipitation (ChIP) was performed according to a protocol published by Agilent technologies (Santa Clara, CA, http://www.agilent.com), with the following modifications. Briefly, the cells were fixed by 11% formaldehyde solution for 10 min. After harvesting the fixed cells, the cells were lysed in lysis buffer, and sonicated using a Misonix XL2020 sonicator (MISONIX Inc., Farmingdale, NY, USA, http://misonix.com) until the DNA fragments were 200–600 base pairs in length. Three percent of the total volume was stored as input at −20 °C, until use. Immunoprecipitation was performed overnight at 4 °C with anti-H3K18Ac (Abcam, ab1191, 5 µg). DNA/beads were washed with a low salt buffer (20 mM Tris-HCl pH 7.4, 150 mM NaCl, 2 mM EDTA, 0.1% sodium dodecyl sulfate (SDS), 1% Triton X-100) once, and then further washed with a high-salt buffer (20 mM Tris-HCl pH 7.4, 400 mM NaCl, 2 mM EDTA, 0.1% SDS, 1% Triton X-100) once before washing with a RIPA buffer. Immune complexes were disrupted with direct elution buffer (50 mM Tris-HCl pH8.0, 10 mM EDTA, 1% SDS) and the covalent links between immunoprecipitates and input chromatin were disrupted by incubation overnight at 65 ℃. DNA was further incubated with RNaseA and proteinase K (Nacalai Tesque), purified by phenol extraction, and ethanol precipitated. DNA pellets were dissolved in Tris-EDTA (TE) buffer (10 mM Tris-HCl, 1 mM EDTA pH8.0). The co-immunoprecipitated DNA was detected via quantitative PCR by using primers for *GBX2* and *AJAP1* genes (Table S5). Three independent experiments were performed.

### 4.10. Statistical Analysis

Statistical analyses were performed using unpaired one- or two-tailed Student’s *t*-tests for single comparisons, and repeated-measures analysis of variance (ANOVA) with the Tukey–Kramer multiple comparison test. Data were considered statistically significant if the *p* value was <0.05. Microsoft Excel 2016 [v. 16.16.4 (181110)] was used for statistical analysis.

## Supplementary Materials

Supplementary materials can be found at https://www.mdpi.com/1422-0067/21/13/4772/s1. **Figure S1.** Diagram depicting the cell culture and neuronal differentiation schedule. hfNSCs are maintained in an undifferentiated state by medium including bFGF and EGF. After the cells reach confluence, the medium is replaced with neuronal differentiation medium. Accompanying the induction of differentiation, the cells are treated with Bangle extract. **Figure. S2.** *cis/trans*-Banglene promote the neuronal differentiation of hfNSCs. (A, B) Cells treated with or without 1 µM *cis/trans*-Banglene were stained with antibodies against DCX (green), CTIP2 (red), and SOX2 (blue) 7 days after induction of differentiation. *Insets*: Hoechst nuclear staining of each field. Scale bar: 50 µm. The graphs indicate the percentage of total cells that were DCX-, CTIP2-, or SOX2-positive. The values shown are the means ± SD. (*N* = 5, *t*-test, * *p* < 0.05, ** *p* < 0.01, *** *p* < 0.005, **** *p* < 0.001). **Figure. S3.** Promotion of neurite outgrowth of Banglene-treated cells disappears at 7 days after neuronal differentiation of hfNSCs. Neurite length of immature neurons (βIII tubulin+) 7 days after induction of differentiation is shown as mean ± SEM. (*N* = 60, **p* < 0.05). **Figure. S4.** Assessment of cell death and cell division in cells treated with Bangle extract and *cis/trans*-Banglene. (A) hfNSCs treated with Bangle extract and *cis/trans*-Banglene were exposed to PI for 10 min 4 days after the induction of differentiation. PI-incorporated cells were directly observed (red). (B, C) hfNSCs treated with Bangle extract and *cis/trans*-Banglene were exposed to EdU, which labels proliferating cells, for 120 min under undifferentiated state (B) or 4 days after induction of differentiation (C). The Click-iT reaction revealed EdU staining (green). *Insets*: Hoechst nuclear staining of each field. Scale bar: 50 µm. The percentage of cells that were PI- and EdU-positive was calculated. Data represent mean ± SD. (*N* = 4). **Figure. S5.** Treatment with the WNT signaling activator CHIR99021 promotes β-catenin nuclear translocation. (A) Western blot analysis of nuclear β-catenin expression in 10 µM CHIR99021 and 10 ng/mL Bangle extract-treated hfNSCs. (B) Western blot analysis of nuclear and cytoplasmic β-catenin expression in 10 ng/mL and 10 µg/mL Bangle extract-treated hfNSCs. LaminB1 and GAPDH were used as nuclear and cytoplasmic fraction markers, respectively. The high exposure image in β-catenin was showed. **Figure. S6.** Treatment with Bangle extract induces the reduction of H3K18Ac enrichment in the proximity to *AJAP1* gene. ChIP assays for hfNSC at 2 days after induction of differentiation were performed using anti-H3K18Ac antibody, and control IgG, followed by real-time PCR with the primer sets in the proximity of *AJAP1* gene. The relative amounts are the means ± SEM compared with those for H3K18Ac in DMSO treatment. (*N* = 3, *t*-test, * *p* < 0.05, ** *p* < 0.01, *** *p* < 0.005). **Figure. S7.** The expression levels of *HDAC9* was not significantly increased by *c*-Banglene treatment. Quantitative RT-PCR analysis of the cells 2 days after the induction of differentiation. Data represent mean ± SEM, when compared to control cells. (*N* = 3, *t*-test, NS: not significant). **Table S1.** Common upregulated genes by treatment with Bangle extract and *c*-Banglene. **Table S2.** List of differentially expressed genes. **Table S3.** Primary and secondary antibody specifications. **Table S4.** List of gene specific primers for qRT-PCR. **Table S5.** List of gene specific primers for ChIP assay.

## Abbreviations

NSCs	Neural stem cells
SAMP8	Senescence Accelerated Mouse-Prone 8
APP	amyloid precursor protein
PI	propidium iodide
EdU	5-ethynyl-2′-deoxyuridine
DMSO	dissolved in dimethyl sulfoxide
DCX	doublecortin
TBR2	T-box brain protein 2
DLX2	Distal-less homeobox 2
AJAP1	adherens junctions associated arotein 1
GBX2	gastrulation brain homeobox 2
GAPDH	glyceraldehyde-3-phosphate dehydrogenase
H3K18Ac	acetylated histone H3 lysine 18
HDAC9	histone deacetylase-9
ChIP	chromatin immunoprecipitation
NPCs	neuronal progenitor cells
GSK3β	glycogen synthase kinase 3β
LSD1	lysine specific demethylase 1
FAD	flavin adenine dinucleotide
GADD45G	Growth Arrest And DNA Damage Inducible Gamma
