# Supplementary material for: Indonesian Ginger (Bangle) Extract Promotes Neurogenesis of Human Neural Stem Cells through WNT Pathway Activation
Source: Int J Mol Sci. 2020 Jul 5;21(13):4772. doi: 10.3390/ijms21134772 (PMC7369972; doi:10.3390/ijms21134772)
Supplement: Supplementary file 1 [file ijms-21-04772-s001.zip › Supplemental Figures and Tables_IJMS.pdf]

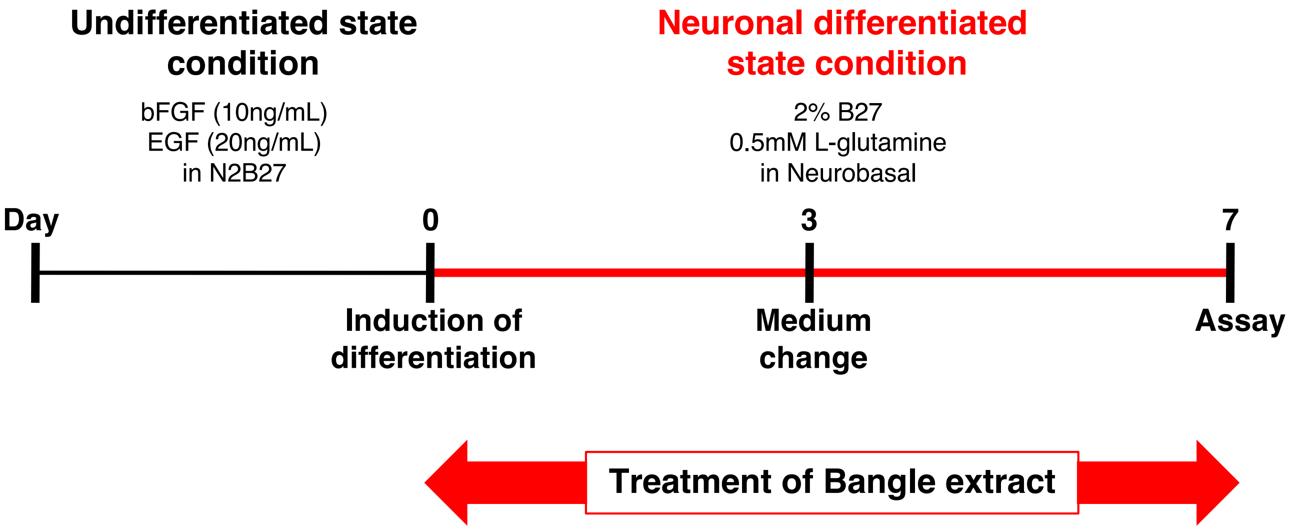

Fig. S1 Diagram depicting the cell culture and neuronal differentiation schedule

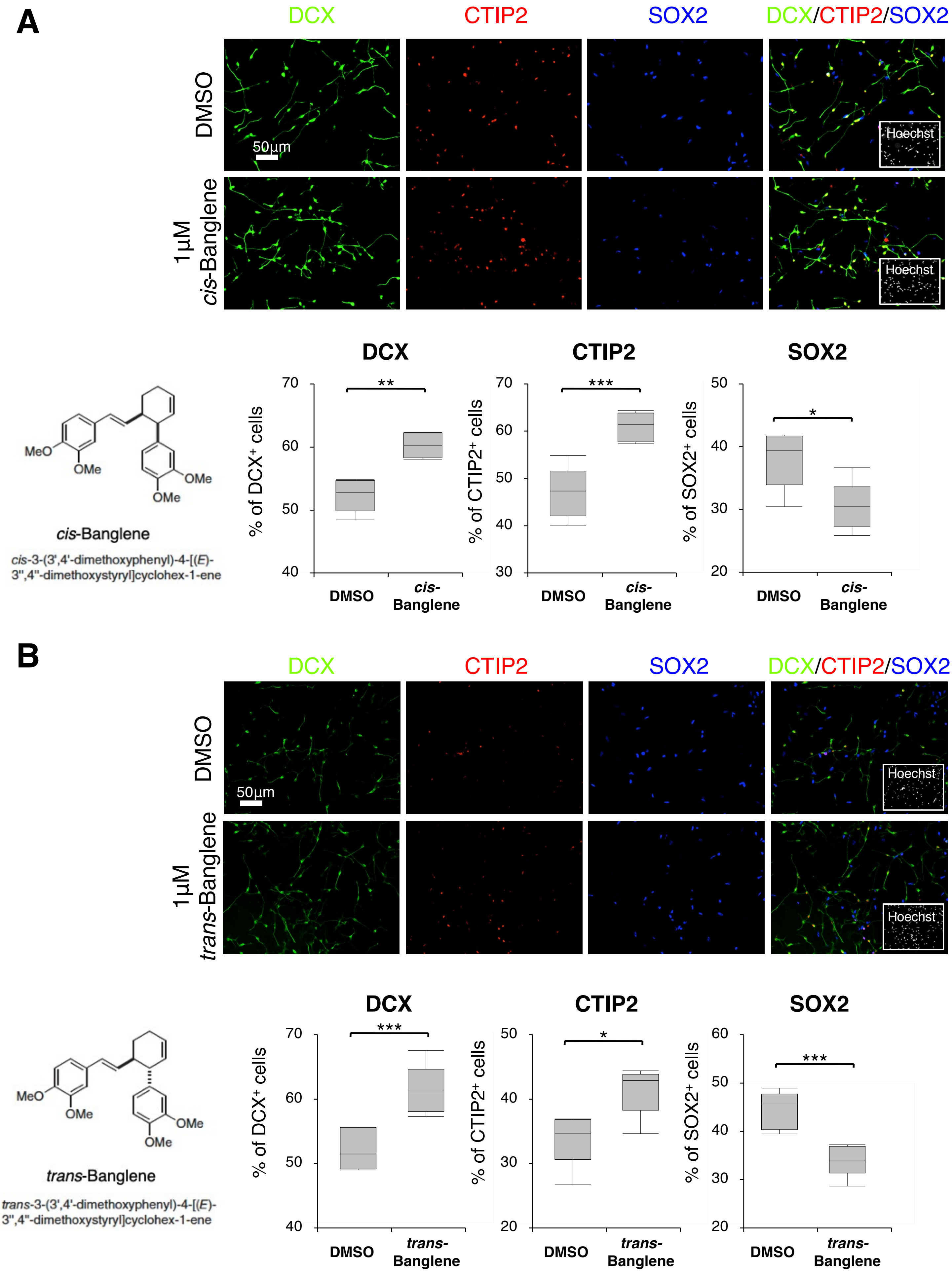

Fig. S2 *cis*-, *trans*-Banglene promote the neuronal differentiation of hfNSCs

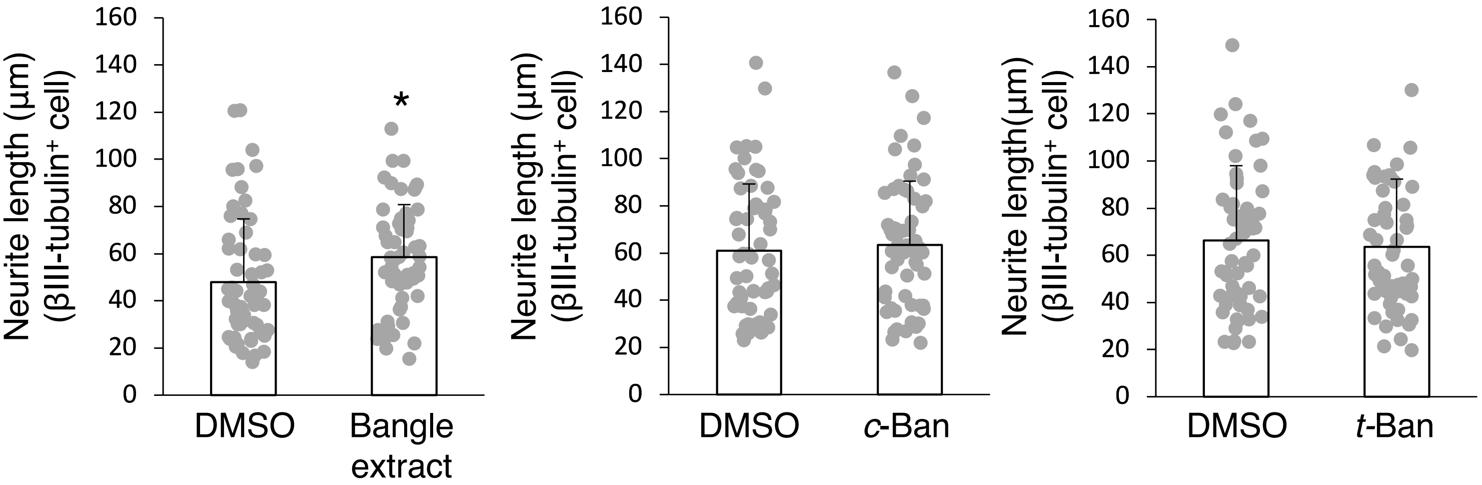

Fig. S3 Promotion of neurite outgrowth of Banglene-treated cell disappears at 7 days after neuronal differentiation of hfNSCs

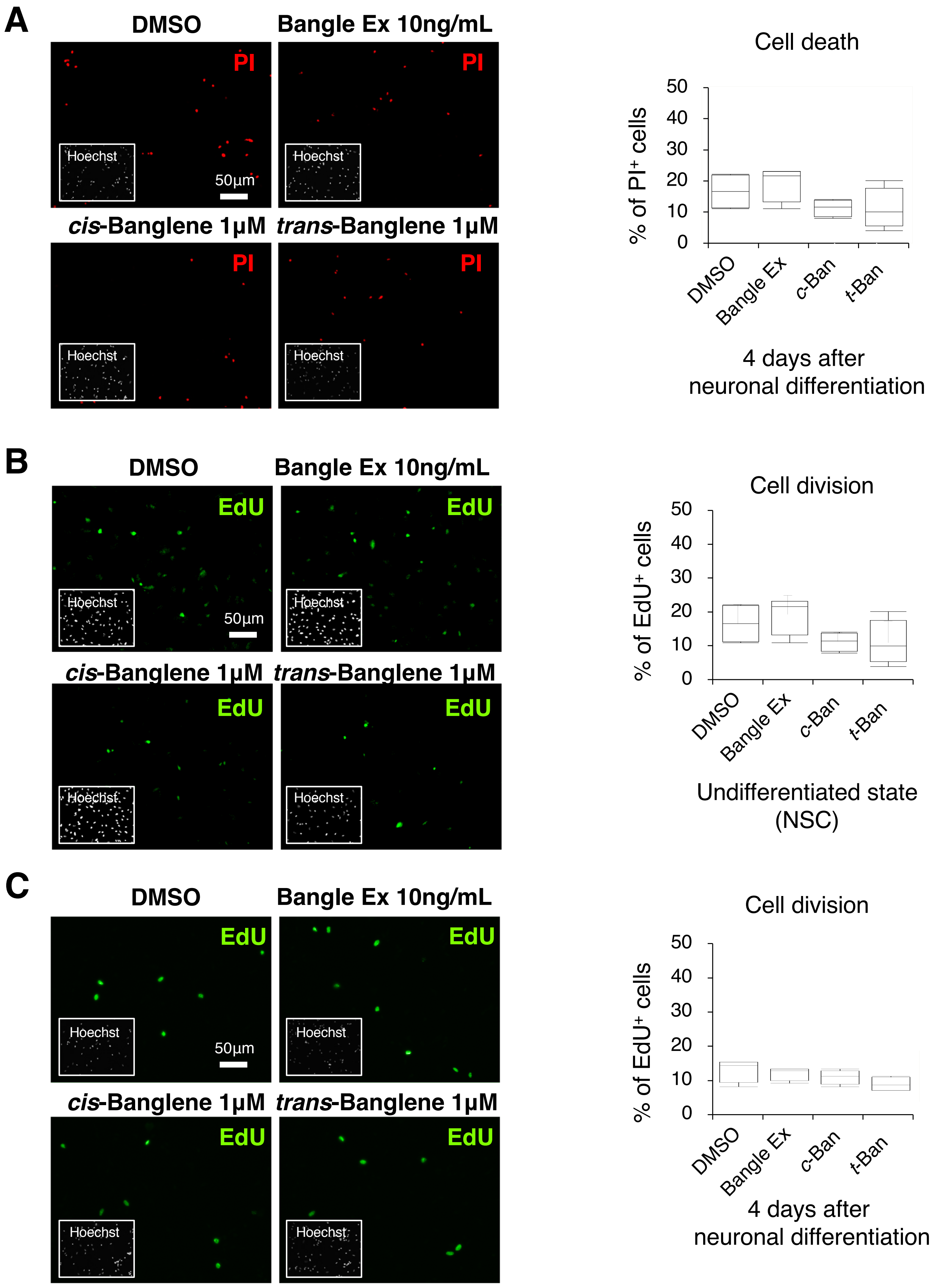

Fig. S4 Assessments of cell death and cell division in cells treated with Bangle extract and Banglene

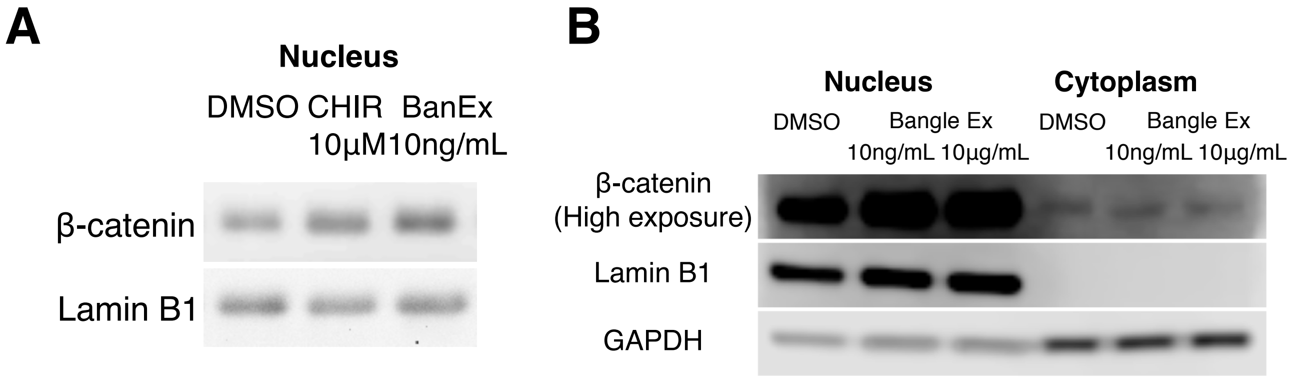

Fig. S5 Treatment with the WNT signaling activator CHIR99021 promotes β-catenin nuclear translocation.

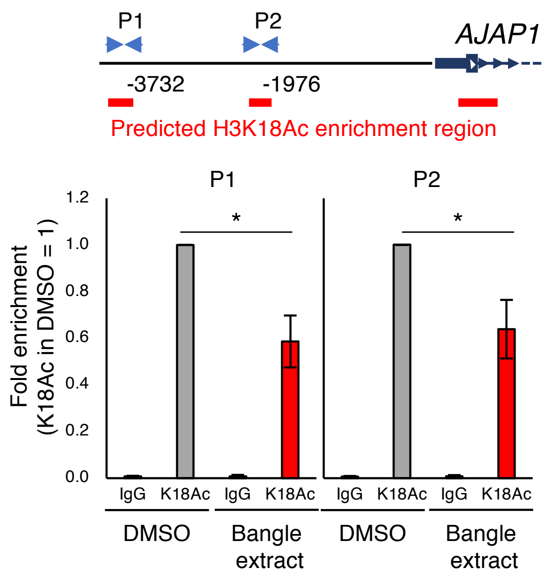

Fig. S6 Treatment Bangle extract decreased H3K18Ac enrichment in the proximity to *AJAP1* gene.

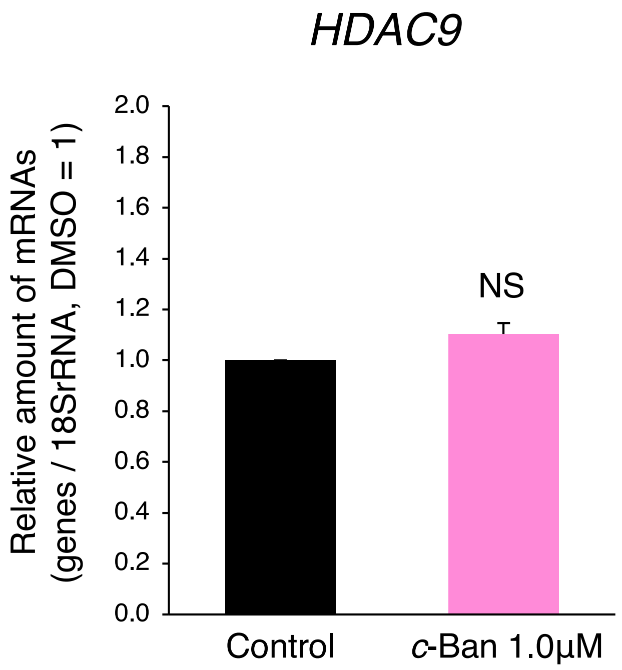

Fig. S7 The expression levels of *HDAC9* was not significantly increased by *c*-Banglene treatment.

**Common Up-regulated Genes**<br>(Bangle Ex/DMSO > 1.3-fold; *c*-Ban/DMSO > 1.3-fold; P< 0.05)					
ACSL6	CRB1	GADD45G	KCNIP3	MFNG	RMST
ATP2B4	DACH1	GCNT1	LAMP5	MOB3B	SERPING1
BEST3	**DCX**	GFAP	LINC00240	MTRR	SHD
C11orf87	**DLX2**	GPR88	LIX1	**MYCN**	SLMAP
CABP7	EFNA1	HELT	lnc-CD99L2-1	NMU	SNCAIP
CASC15	**EOMES**	HES5	lnc-DYNC1I2-1	PCDH8	TESC
CASZ1	FAM46B	HYDIN	lnc-SLC7A7-1	PRODH	TOX2
CDON	FEZF2	IGFBPL1	LOC101929759	RAB3C	VCAM1
CNGA3	FRMD4B	KCNG1	LOC115110	RGS16	

**Common Down-regulated Genes**<br>(Bangle Ex/DMSO < 0.7-fold; *c*-Ban/DMSO < 0.7-fold; P< 0.05)	
**AJAP1**	LGI2
CABLES1	lnc-MLXIP-1
**GBX2**	LOC100130938
KCNA2	SPRY4

Table S1 Common up- and down-regulated genes by treatment with Bangle extract and *c*-Banglene

Table S2. List of differentially expressed genes.
See excel file Table S2

	Antibody Target	Species	Supplier	Cat.No.	RRID	Dilution
Primary Abs for immunostaining	βIII-tubulin	Mouse	R&D systems	MAB1195	AB_357520	1:5000
	β-catenin	Rabbit	Abcam	ab32572	AB_725966	1:200
	CTIP2	Rat	Abcam	ab18465	AB_2064130	1:1000
	DCX	Chicken	Abcam	ab153668	AB_2728759	1:200
	SOX2	Rabbit	Abcam	ab97959	AB_2341193	1:200
Secondary Abs for immunostaining	CF488 Donkey anti-mouse IgG	Donkey	Biotium	#20014	AB_10853131	1:500
	CF568 Donkey anti-rabbit IgG	Donkey	Biotium	#20098	AB_10853318	1:500
	CF647 Donkey anti-rabbit IgG	Donkey	Biotium	#20047	AB_10853792	1:500
	CF488 Donkey anti-chicken IgY	Donkey	Biotium	#20166	AB_10854387	1:500
	CF568 Donkey anti-rat IgG	Donkey	Biotium	#20092	AB_10855000	1:500
Primary Abs for WB	β-catenin	Rabbit	Abcam	ab32572	AB_725966	1:5000
	LaminB1	Rabbit	MBL	PM064	AB_10693917	1:2000
	H3K18Ac	Rabbit	Abcam	ab1191	AB_298692	1:500
	H3K4me1	Rabbit	Abcam	ab8895	AB_306847	1:500
	Histone H3	Rabbit	Abcam	ab1791	AB_302613	1:5000
	GAPDH	Mouse	EMD Millipore	MAB374	AB_2107445	1:4000
Secondary Abs for WB	HRP Goat anti-Mouse IgG	Goat	Abcam	ab97023	AB_10679675	1:5000
	HRP Goat anti-Rabbit IgG	Goat	Abcam	ab97051	AB_10679369	1:5000

Table S3 Primary and secondary antibody specifications

Gene	Forward primer sequence (5'-3')	Reverse primer sequence (5'-3')
hTBR2	CCACTGCCCACTACAATGTG	TTCCCGAATGAAATCTCCTG
hDCX	GACAGCCCACTCTTTTGAGC	TGGGTTTCCCTTCATGACTC
hDLX2	GCACATGGGTTCCTACCAGT	TCCTTCTCAGGCTCGTTGTT
hN-MYC	CTTCGGTCCAGCTTTCTCAC	GTCCGAGCGTGTTCAATTTT
hAXIN2	CCTGCCACCAAGACCTACAT	CTTCATTCAAGGTGGGGAGA
hEGFR	CAGAGTGATGTCTGGAGCTACG	GGGAGGCGTTCTCCTTTCT
hAJAP1	GTTAGCACAACGGAGCCTTC	GATGATCTGATGGACAGCCA
hGBX2	AAAGAGGGCTCGCTGCTC	GGTCGTCTTCCACCTTTGAC
hHDAC9	GCAACAGGTAATAGGCAAAG	CACTGAACTCAAATCATAGAG
h18SrRNA	GATATGCTCATGTGGTGTTG	AATCTTCTTCAGTCGCTCCA
hβ-actin	TTCTACAATGAGCTGCGTGTG	GGGGTGTTGAAGGTCTCAAA

Table S4 List of gene specific primers for qRT-PCR

Gene	Forward primer sequence (5'-3')	Reverse primer sequence (5'-3')
**hGBX2_ChIP_1**	GATCTGGTGGTGAGGGTGTG	CAGCATAGACTCGCTGATCG
**hGBX2_ChIP_2**	TGTATTTGCCATGGAACCAA	GGACCTACAGCGAGCAACC
**hGBX2_ChIP_3**	GGGATCTCCAGGCTCTATCC	AAGGAAGGAATGGAGCGTTT
**hGBX2_ChIP_4**	ATCCGGAGGAGGAAGCAC	AGCCTGTCGCCTAGGACTG
**hAJAP1_ChIP_1**	AATGGCCTCAGAGAGCTTCA	GTGGAAAGTTCTTCCGGTCA
**hAJAP1_ChIP_2**	GAGAGGCTCTAGGGGACGAT	GACCCTCGGTCTCCTCATCT

Table S5 List of gene specific primers for ChIP assay
